# Antimicrobial activities of Eugenol and Cinnamaldehyde against the human gastric pathogen *Helicobacter pylori*

**DOI:** 10.1186/1476-0711-4-20

**Published:** 2005-12-21

**Authors:** Shaik Mahaboob Ali, Aleem A Khan, Irshad Ahmed, M Musaddiq, Khaja S Ahmed, H Polasa, L Venkateswar Rao, Chittoor M Habibullah, Leonardo A Sechi, Niyaz Ahmed

**Affiliations:** 1 Department of Microbiology, Osmania University, Hyderabad – 500 007, India; 2 Centre for Liver Research and Diagnostics, Deccan Medical College & Allied Hospitals, Kanchanbagh, Hyderabad – 500 058, India; 3 Pathogen Evolution Group, Center for DNA Finger printing and Diagnostics, Nacharam, Hyderabad- 500 076, India; 4 ISOGEM Collaborative Network on Genetics of Helicobacters, The International Society for Genomic and Evolutionary Microbiology, Sassari, Italy; 5 Department of Microbiology, Shri Shivaji College, Akola, India; 6 Dipartimento di Scienze Biomediche, Sezione Microbiologia sperimentale e clinica, Facoltà di Medicina, Universiti degli studi de Sassari, viale S. Pietro, 07100 Sassari, Italy

## Abstract

**Background:**

Eradication of *Helicobacter pylori *is an important objective in overcoming gastric diseases. Many regimens are currently available but none of them could achieve 100% success in eradication. Eugenol and cinnamaldehyde that are commonly used in various food preparations are known to possess antimicrobial activity against a wide spectrum of bacteria.

**Aim:**

The present study was performed to assess the *in vitro *effects of eugenol and cinnamaldehyde against indigenous and standard *H. pylori *strains, their minimum inhibitory concentrations (MICs) and time course lethal effects at various pH.

**Methods:**

A total of 31 strains (29 indigenous and one standard strain of *H. pylori *ATCC 26695, one strain of *E. coli *NCIM 2089) were screened. Agar dilution method was used for the determination of drug sensitivity patterns of isolates to the commonly used antibiotics and broth dilution method for the test compounds.

**Results:**

Eugenol and cinnamaldehyde inhibited the growth of all the 30 *H. pylori *strains tested, at a concentration of 2 μg/ml, in the 9th and 12th hours of incubation respectively. At acidic pH, increased activity was observed for both the compounds. Furthermore, the organism did not develop any resistance towards these compounds even after 10 passages grown at sub-inhibitory concentrations.

**Conclusion:**

These results indicate that the two bioactive compounds we tested may prevent *H. pylori *growth *in vitro*, without acquiring any resistance.

## Introduction

*Helicobacter pylori *was first discovered in 1982 and was subsequently found to be an important etiological agent for gastritis [1], peptic ulcers [2] and gastric malignancy [3]. It is estimated that one-half of the world's population is infected with *H. pylori *[4]. Numerous clinical evidences reveal that eradication of *H. pylori *results in improvement of gastritis and drastically decreases the rate of relapse of gastric and duodenal ulcers [5,6]. *H. pylori *carriage rates are about 80–90% in developing countries [7], with a high risk of gastric cancer and antibiotic regimens against *H. pylori *are frequently ineffective in such populations [8]. Besides this, undesirable side effects [9], noncompliance among the patients [10] and the cost of the antibiotic regimens [11] are few other factors for their ineffectiveness. Hence there is a need to develop alternative approaches to suppress/cure the infection.

Since ancient times, spices and condiments have been considered indispensable in the culinary arts, as they are used to flavor foods. Also these spices were recognized for their physiological and medicinal properties. It is presumed that the broad-spectrum effectiveness of these spices [12-14] may provide a suitable basis for new anti *H. pylori* therapies. Some of the epidemiological studies suggest an inverse relationship between the occurence of cancer of stomach (for which *H. pylori* infection is a known risk factor) and the intake of the allium vegetables [15,16]. There are reports on *in vitro *antimicrobial activity of many spice extracts (and not their active principles) on *H. pylori *[17,18]. But active principles of garlic and garlic components have been tried [11,19].

Eugenol, a phenolic compound is the main component of clove (*Eugenia caryophillis*) oil. Cinnamaldehyde is an aromatic aldehyde and main component of bark extract of cinnamon (*Cinnamomum verum*). Both these compounds are present in essential oils of many plants and are proved to be active against many pathogenic bacteria [20,21], fungi [21,22] and viruses [14]. There is a paucity of information on the effects of these bioactive compounds on *H. pylori*. Therefore, the present study was undertaken to evaluate the efficacy of eugenol and cinnamaldehyde as anti *H. pylori *agents.

## Materials and methods

### *H. pylori *strains

Thirty *H. pylori *strains and one *E. coli *strain (NCIM2089) were used in the study. Twenty-nine strains that were designated as MS-1 to MS-29 were isolated from gastric biopsy specimens (15 from duodenal ulcer, 10 from non ulcer dyspepsia and 4 from gastric ulcer subjects), after informed consents from patients who have undergone upper gastrointestinal endoscopy at Deccan College of Medical Sciences, Hyderabad, India. One strain of *H. pylori *(ATCC26695) was also included.

Primary isolation was performed on Brucella agar (Becton, Dickinson & Co, Sparks, USA), supplemented with 7% (V/V) sheep blood and antibiotics (Vancomycin, 6 mg/L; Amphoteracin B, 3 mg/L; Polymixin B, 2500 IU/L; Hi-Media, Mumbai, India). Final pH of the media was adjusted to 7.2. *H. pylori *was identified by its typical colony morphology, Gram staining, microaerophilic growth at 37°C and by other biochemical tests such as oxidase, catalase and urease. These strains were preserved at -80°C in brucella broth (Becton, Dickinson & Co, Sparks, USA) containing 10% (V/V) fetal calf serum (FCS) and 20% (V/V) glycerol.

### Bioactive compounds

Commercial preparations of bioactive compounds such as eugenol and cinnamaldehyde were used in our experiments. All the strains were seeded on Brucella agar supplemented with 7% sheep blood and grown for 48 hours under microaerobic conditions. Bacterial growth was harvested from the plates and resuspended in sterile saline. The inoculum was prepared to contain ~10^8 ^CFU/ml by adjusting the suspension to match the McFarland No-4 turbidity standard [23].

### Antibacterial activity of standard antibiotics

Minimum inhibitory concentrations (MICs) for antibiotics like metronidazole, amoxycillin and clarithromycin were determined by agar dilution method, using two fold increments (0.016 to 256 μg/ml) on Mueller Hinton agar (MHA) plates supplemented with 7% sheep blood and incubated for 3 days at 37°C in a microaerophilic cabinet. MICs were determined as the lowest concentration of the antibiotics inhibiting visible growth [24] using ~10^6 ^CFU of inoculum.

### Antibacterial activity of eugenol and cinnamaldehyde

#### Primary screening

The Kirby-Bauer disk diffusion susceptibility test was used to check the activity of the bioactive compounds on *H. pylori*. The inoculum prepared as described previously was inoculated onto MHA supplemented with 7% sheep blood and bioactive compound discs (prepared by loading the respective compounds in variable concentration on to the sterile filter paper discs) were placed on to the plates with the sterile dispenser and incubated in microaerobic atmosphere at 37°C for 3 days.

#### Minimum inhibitory concentration (MIC) of bioactive compounds

As there was no established method to determine the antibacterial activity of bioactive compounds against *H. pylori*, serial two fold dilutions of eugenol and cinnamaldehyde were made in a 5 ml Brucella broth preparation (pH-7) with 0.1% dimethyl sulfoxide (DMSO; that enhances compound solubility) and supplemented with 2.5% fetal calf serum (FCS). Cotton-wool plugged 25 ml conical flasks of Brucella broth prepared as above, were inoculated with *H. pylori *(~10^6 ^CFU/ml)[11]. The flasks were incubated for 3–5 days in a microaerophilic cabinet with shaking at ~150 rpm. Following incubation, 100 μl aliquots of the medium were plated onto brucella agar plates supplemented with 7% sheep blood to determine the viable counts.

#### Time Course

Time course viability studies of the bioactive compounds were set up in the same way as the above MIC tests, but the broths were sampled hourly over the first 4 h and then again at 6, 9, 12, 24, 48 and 72 h to check the viable count. *H. pylori *ATCC26695 was used as the inoculum for the study.

For comparison, known antibiotic such as amoxycillin was used and *E. coli *(NCIM2089) was used as a standard bacterial control other than *H. pylori*.

### Effects of bioactive compounds on *H. pylori *morphology

To check the effect of bioactive compounds on the morphology of *H. pylori*, slides were prepared from the above samples of first four hours, stained with Giemsa and observed under oil immersion.

### Measurement of bactericidal activity at acidic pH

The effects of pH on the antibacterial activities of eugenol and cinnamaldehyde were done as reported previously [25,26]. The pH of the buffer was adjusted to 4.00 using 100 mM citrate buffer (pH 5.2) supplemented with 10 mM urea and the pH was adjusted to 7.00 using 10 mM phosphate buffer (pH 6.8). *H. pylori *ATCC26695 (~10^7 ^CFU/ml) was inoculated into 5 ml of the medium with 0.1% DMSO to which various concentrations of the compounds were added. All the flasks were incubated at 37°C. Twenty μL of the samples were taken at 0, 15, 30 and 60 minutes after inoculation to enumerate the number of viable cells.

### Test for development of resistance to bioactive compounds

To investigate the development of resistance, five strains i.e. ATCC26695, MS-27 (sensitive to all the three antibiotics), MS-1 (resistant to all the three antibiotics), MS-8 (resistant to metronidazole, clarithromycin and sensitive to amoxycillin), and MS-15 (resistant only to metronidazole), were grown on agar at sub inhibitory concentrations (0.25 and 0.5 μg/ml) of eugenol and cinnamaldehyde for 10 passages. All the experiments stated above were repeated three times.

## Results

### Antibacterial activity against *H. pylori*

MICs of antibiotics for *H. pylori *were determined. Strain ATCC26695 was sensitive (MIC < 0.016 μg/ml) to all the three drugs tested. Twenty (66.7%) clinical isolates (MS1-8, 10, 12, 14 – 17, 20, 23 – 26 and MS28) from our laboratory were resistant to metronidazole (MICs > 8 μg/ml), 2 (6.7%) clinical isolates (MS1 & 8) were resistant to clarithromycin (MICs > 2 μg/ml) and 1 (3.3%) clinical isolate (MS1) was resistant to amoxycillin (MIC > 0.5 μg/ml) [27] (Table 1).

**Table 1:** Sensitivity of *H. pylori *strains (n = 30) to different antibiotics

**Antibiotic**	**Sensitive (%)**	**Resistance (%)**	**Range (μg/ml)**	**MIC^27 ^(μg/ml)**
Metronidazole	10 (33.3)	20 (66.7)	<0.5 – >256	>8
Clarithromycin	28 (93.3)	2 (6.7)	<0.5 – >256	>2
Amoxycillin	29 (96.7)	1 (3.3)	<0.5 – >256	>0.5

In the preliminary screening, both the compounds gave approximately 15 mm inhibition zones to all the clinical isolates and standard strain (*H. pylori *ATCC26695). This *in vitro *antimicrobial effect of the bioactive compounds demonstrated that both the compounds were capable of completely inhibiting the growth of all the strains (both sensitive & resistant) at a concentration of 2 μg/ml. For *E. coli *(NCIM-2089), MIC of eugenol and cinnamaldehyde was 1 μg/ml.

### Time course

**Figure 1:**
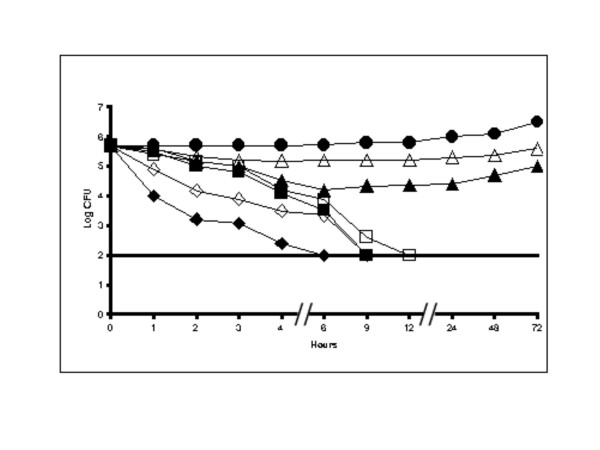
**Time course viability studies of eugenol and cinnamaldehyde with *H. pylori *ATCC26695**. ATCC26695 was exposed to eugenol at concentrations of (◆) 4 μg/ml, (■) 2 μg/ml, (▴) 1 μg/ml, (●) control, and to cinnamaldehyde (◊) 4 μg/ml, (▫) 2 μg/ml, (△) 1 μg/ml. Detection limit was shown with a straight line. The controls were maintained with 0.1% DMSO.

As both the compounds were active at a concentration of 2 μg/ml, we examined the killing curve time course of both the compounds at different concentrations i.e. 1 μg/ml, 2 μg/ml and 4 μg/ml. Eugenol completely inhibited the growth of *H. pylori *ATCC 26695 at 2 μg/ml concentration in 9 hours of incubation. The growth was completely inhibited in only 6 hours when the MIC was doubled (4 μg/ml). Some inhibition of growth was observed at the MIC of 1 μg/ml, where up to 6^th ^hour a decrease of ~1.6 Log was observed then afterwards there was no effect of the compound on the bacterium (Figure 1). Cinnamaldehyde inhibited ATCC26695 totally at 2 μg/ml in 12 hours and in 9 hours at double the MIC (4 μg/ml). However, at half of the MIC (1 μg/ml), a little effect was observed up to the 3^rd ^hour and there was a decrease of 0.6 Log with no subsequent inhibitory effect (Figure 1).

**Figure 2:**
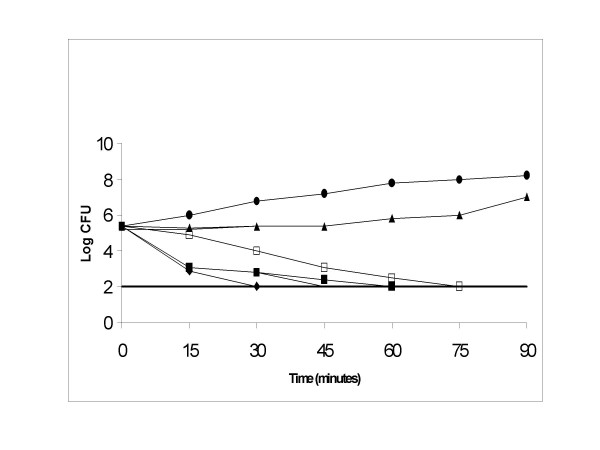
**Effects of eugenol and cinnamaldehyde on the growth of *E. coli *NCIM-2089**. *E. coli *was exposed to eugenol at concentrations of (◆) 2 μg/ml, (■) 1 μg/ml, (▲) 0.5 μg/ml, (●) control, and to cinnamaldehyde at concentrations of (◊)2 μg/ml, (□) 1 μg/ml, (△) 0.5 μg/ml. Detection limit was shown with a straight line. The controls were maintained with 0.1% DMSO.

The time course lethal action of the bioactive compounds on *E. coli *(NCIM-2089) was studied at three different concentrations (1 μg/ml, 2 μg/ml & 0.5 μg/ml). At 1 μg/ml, eugenol inhibited *E. coli *after 1 hour of incubation whereas cinnamaldehyde inhibited *E. coli *after 75 minutes of incubation (Figure 2).

**Figure 3:**
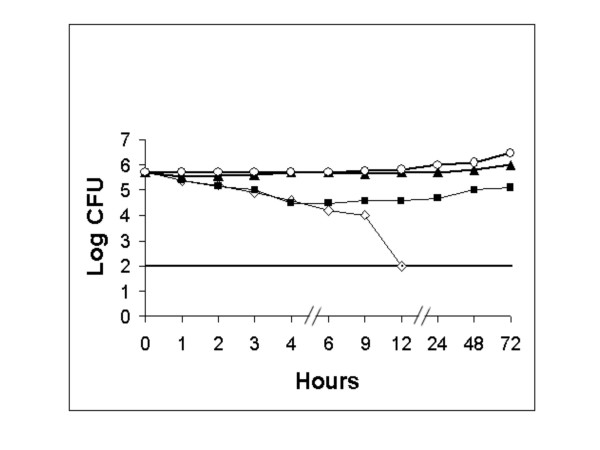
**Effect of amoxicillin on the growth of *H. pylori *(ATCC26695)**. ATCC26695 was exposed to at concentrations of (◊) 1 μg/ml, (■) 0.1 μg/ml, (▴) 0.01 μg/ml, (○) control. Detection limit was shown with a straight line.

For comparing the efficacy of our bioactive compounds against *H. pylori*, we studied the effect of a common antibiotic (amoxycillin) at different concentrations such as 1 μg/ml, 0.1 μg/ml and 0.01 μg/ml with an MIC of < 0.016 μg/ml. There was no inhibition of *H. pylori *seen at the concentration of 0.01 μg/ml. But inhibition was noticed at a concentration of 0.1 μg/ml up to the 4^th ^hour of incubation. However, the bacterium was completely inhibited at a concentration of 1 μg/ml, by the 12^th ^hour of incubation (Figure 3).

### *H. pylori *morphology

Microscopic observation of slides revealed that at high concentrations of both the bioactive compounds (4 μg/ml) and after 4 hours of incubation, ~70% of the cells assumed coccoid forms with fewer spiral shaped organisms.

### Effect of the bioactive compounds on the growth of *H. pylori *in buffers at acidic pH

**Figure 4:**
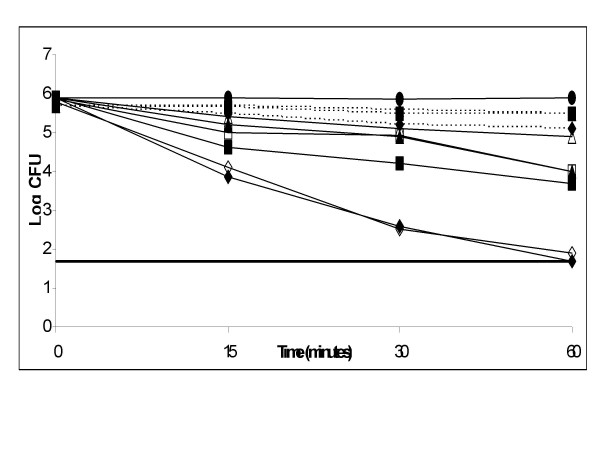
**Effect of eugenol and cinnamaldehyde on the growth of *H. pylori *(ATCC26695) at low pH**. ATCC26695 was exposed to eugenol at pH4 (solid lines) and pH7 (dotted lines) at concentrations (◆ or ◊) 4 μg/ml, (■ or □) 2 μg/ml, (ß or △) 1 μg/ml, (●) control. Detection limit was shown as a straight line. The controls were maintained with 0.1% DMSO.

The viability of strain ATCC 26695 at acidic pH was investigated using eugenol and cinnamaldehyde at various concentrations (2 μg/ml, 4 μg/ml and 1 μg/ml). As shown in Figure 4, both the compounds revealed increased activity at acidic pH (pH-4.0) and a dose dependant bactericidal activity was also observed. At a concentration of double the MIC (4 μg/ml), eugenol was found to completely inhibit the bacteria at about 1 hour of incubation. Cinnamaldehyde also revealed bactericidal activity in the similar manner but did not inhibit the organism totally (after one hour of incubation there was a difference of 0.2 Log). For both the compounds, when compared at acidic and neutral pH, we found a Log difference of 1.5 after one hour of incubation at MIC of 2 μg/ml.

### Test for resistance to eugenol and cinnamaldehyde

We investigated whether *H. pylori *would acquire resistance to the bioactive compounds. Selected *H. pylori *strains (ATCC26695, MS-27, MS-1, MS-8 and MS-15) did not acquire resistance to both the bioactive compounds at sub inhibitory concentrations (0.25 and 0.5 μg/ml) even after 10 passages.

## Discussion

Eradication of *H. pylori *with monotherapy is difficult and often results in poor clinical outcome [28]. Presently a proton pump inhibitor (PPI) based triple therapy is being used. Though it is having a success rate of 80 to 90% [29], problems like treatment failure and contraindications for some patients are common. Furthermore, the major problem in treating *H. pylori *infection is rapidly emerging drug resistance during treatment with various antibiotics [30]. Because of the prevalence of antibiotic-resistant *H. pylori *strains, the search for safe and effective non-antibiotic agents is essential.

In Indian traditional medical system, a number of plants and plant products are known to possess potent medicinal properties, suggesting that plants, plant products and their extracts may be useful for specific medical conditions. Hence in our effort to identify newer compounds, which can inhibit *H. pylori *growth, we tested eugenol and cinnamaldehyde. These compounds have already been found to inhibit 10 different multi drug resistant pathogenic bacteria such as *E. coli*, *Staphylococcus*, *Proteus*, *Klebsiella*, *Enterobacter*, and *Pseudomonas *isolated from human subjects [20,21]. In our studies, eugenol and cinnamaldehyde completely inhibited all the strains (both sensitive & resistant) at a concentration of 2 μg/ml. This inhibitory concentration is very less when compared against the previously reported MICs {allicins: 6–12 μg/ml, garlic oil: 8–32 μg/ml [11] and ajoenes: 10–25 μg/ml [31]}. Our compounds also proved to be more potent than thiosulfinate {40 μg/ml [19]}, vinyldithiins {<100 μg/ml [31]}, epigallocatechin gallate {50–100 μg/ml [32]} and garlic powder {250 μg/ml [11]}.

In time course viability studies, we found that eugenol and cinnamaldehyde inhibited *H. pylori *ATCC26695 at 2 μg/ml concentration within 9 and 12 hours of incubation respectively. Highly potent effects of some essential oils of lemon grass on *H. pylori *have been previously described [26]. However, our observations may be more accurate with the fact that we used active principles and not the extracts or oils as used by Ohno et al., [26]. These essential oils may contain unknown substances that might provide synergistic, inhibitory effects. Eugenol in our studies was found to be a potent antimicrobial than the essential oils of Ohno and colleagues [26] whereas the effect of cinnamaldehyde was approximately equivalent to the oils used by them. Further, our compounds were more potent than the common antibiotic amoxycillin, which inhibited *H. pylori *ATCC26695 at 12^th ^hour (similar to cinnamaldehyde) whereas eugenol inhibited microbial growth at the 9^th ^hour of incubation itself, at its minimum inhibitory concentration. Amoxycillin was found to completely inhibit bacteria at a higher concentration (1 μg/ml), than it's minimum inhibitory concentration (0.016 μg/ml) (Figure 3). As *H. pylori *resides in the stomach, where pH levels are lower than 3.0, it is important to check the activity and stability of a compound at acidic pH. In our study, *H. pylori *isolates survived at pH 4.0, despite some reports showing *H. pylori *survival at pH-3.0 [25], and bactericidal effects of the bioactive compounds were found to be stronger even at low pH. Several agents that have bactericidal effects against *H. pylori *are dependent on pH (for example, ecabet sodium is active at acidic pH [25] and tea cathechin is active at alkaline pH [33]).

Furthermore, we found that the organism did not develop any resistance to the test compounds even after 10 successive passages grown at sub-inhibitory concentrations (0.25 and 0.5 μg/ml) [21], whereas *H. pylori *strains acquired resistance to amoxycillin and clarithromycin after 10 sequential passages as reported previously [26,34]. In addition, these bioactive compounds had equivalent bactericidal activities against both the susceptible and resistant *H. pylori *strains.

Eugenol and cinnamaldehyde form a part of regular diet in tropical countries since ages. Many reports suggest these compounds are nontoxic at doses being consumed [34], and they are known for their antimicrobial and antioxidant properties [21,36]. Moreover, to heal peptic ulcer, or to prevent the oxidative stress, prescribing antioxidants is quite a normal practice these days with the medical practitioners. As both these compounds are natural antioxidant [37], their benefits could be multiple. Two animal studies carried out in the past suggest that an extract of cinnamon bark taken orally may help prevent stomach ulcer [36] and one study described eugenol to be both anti-inflammatory and antiulcerogenic [38]. It was used as early as 1950 for treating ulcer disease [39]. Given all the advantages we observed for the two bioactive compounds tested, our data should not be construed as certification of the compounds in clinical application until their stability, specificity and overall efficacy is established in different laboratories and against different bacterial strains. Further studies are therefore clearly needed in the context of reproducibility of these findings under different laboratory conditions.

## Conclusion

The data obtained in this study show that eugenol and cinnamaldehyde at 2 μg/ml produce significant decrease of viability of the organism *H. pylori*, irrespective of the strain. Activity of these compounds at low pH levels may help them achieve their efficacy in an environment such as human stomach. We trust these findings are encouraging in view of growing treatment failures and antibiotic resistance in the area of *H. pylori *management. Additional *in vivo *studies and clinical trials would be needed to justify and further evaluate the potential of these compounds as anti *H. pylori *agents.
